
JOURNAL INFORMATION
==============================
NLM Title Abbreviation: Wirtschaftsdienst
Journal ID: Wirtschaftsdienst
NLM Journal ID: 101086612

Wirtschaftsdienst (Hamburg, Germany : 1949)

ISSN: 0043-6275

ARTICLE INFORMATION
==============================
PMCID: PMC8800394
PMID: 35125545
DOI: 10.1007/s10273-022-3081-z
Article ID: 3081
Article version: 1
Subjects: Kommentare

Rente: Nachholfaktor ist zurück — gut so?

Pension: Catch-up factor is back — a good thing?

Werding Martin martin.werding@ruhr-uni-bochum.de

grid.5570.7 0000 0004 0490 981X Lehrstuhl für Sozialpolitik und öffentliche Finanzen, Ruhr-Universität Bochum, Universitätsstr. 150, 44801 Bochum, Deutschland
Electronic publication date: 2022 Jan 29
Print publication date: 2022
Volume: 102
Issue: 1
First page: 5
Last page: 5
Copyright: © Der/die Autor:in 2022
License: Open Access: Dieser Artikel wird unter der Creative Commons Namensnennung 4.0 International Lizenz veröffentlicht (https://creativecommons.org/licenses/by/4.0/deed.de).
Open Access wird durch die ZBW — Leibniz-Informationszentrum Wirtschaft gefördert.
License URL: https://creativecommons.org/licenses/by/4.0/

Keywords: H55, J11, J26
issue-copyright-statement: © The Author(s) 2022

==============================

DOI: 10.1007/s10273-022-3081-z
Wirtschaftsdienst, 2022, 102(1), 5
JEL: H55, J11, J26

Rente

### Nachholfaktor ist zurück – gut so?

Die Rentenkommission der letzten Legislaturperiode beriet über langfristig orientierte Reformen bei der Altersvorsorge, ohne griffige Ergebnisse. Wer gehofft hat, dass die neue Bundesregierung dieses Thema weiterverfolgt, erlebte bei der Vorlage des neuen Koalitionsvertrags eine Enttäuschung. Für Kenner:innen der rentenpolitischen Diskussionslage enthielt dieser trotzdem eine kleine Überraschung: Bei der Rentenanpassung zum 1. Juli 2022 soll der Ausgleichs- oder Nachholfaktor wieder aktiviert werden, dessen Anwendung die alte Bundesregierung bis 2026 ausgesetzt hatte. Aufgehoben wird damit eine Sonderregelung zu einer Sonderregelung im Rentenanpassungsrecht, von der vorab wohl niemand geahnt hat, dass sie irgendeine praktische Bedeutung haben würde. Nun dürfte sich die für 2022 absehbare Rentenerhöhung leicht abschwächen, von einem Plus von mehr als 5 % auf etwas über 4 %. Wie ist dieser Schritt einzuordnen?

Die derzeit gültige Formel zur Berechnung der jährlichen Rentenanpassungen sieht kompliziert aus. Letztlich stehen dahinter aber drei einfache Grundideen: *Erstens* folgen die Renten – wie schon seit 1957 – mit einjähriger Verzögerung der Entwicklung der Bruttolöhne der aktiv Versicherten. *Zweitens* wird dabei – mit gewissen Variationen in der genauen Umsetzung seit 1992 – letztlich eine angemessene Nettorelation von Renten und Löhnen angezielt. Daher werden Änderungen der Beitragssätze der Rentenversicherung berücksichtigt, die die Aktiven be- oder entlasten. *Drittens* werden die ungünstigen Effekte des laufenden demografischen Alterungsprozesses für die Rentenfinanzen zwischen Rentner:innen und Aktiven geteilt. Dafür sorgt seit 2005 der „Nachhaltigkeitsfaktor“, durch den die Rentenanpassungen auf Verschiebungen des zahlenmäßigen Verhältnisses von Rentenbeziehenden zu Beitragszahlenden (zusätzlich gewichtet mit der Höhe ihrer Ansprüche bzw. ihrer beitragspflichtigen Entgelte) reagieren.

Neben diesen Standardregeln für die Rentenanpassungen gibt es noch verschiedene Sonderregelungen. So gilt seit 2009 eine „Schutzklausel“, nach der die Renten nicht gesenkt, sondern konstant gehalten werden, wenn die Löhne der Versicherten im Vorjahr gesunken sind. Damit sich das Rentenniveau dadurch nicht dauerhaft erhöht, wird bei Anwendung dieser Klausel allerdings ein Ausgleichsbedarf berechnet, um den sich die Rentenanpassungen verringern, sobald die Löhne wieder steigen. Daraus ergibt sich der Ausgleichs- oder Nachholfaktor, dessen Anwendung 2018 befristet suspendiert wurde. Seither gilt zugleich eine „Haltelinie“ für das Rentenniveau, das in seiner gesetzlichen Definition als „Sicherungsniveau vor Steuern“ bis 2025 48 % nicht unterschreiten darf. Die Aussetzung des Nachholfaktors diente in erster Linie dazu, die dafür erforderlichen Berechnungen nicht unnötig zu komplizieren.

Anschließend geschah, womit damals nicht zu rechnen war. Wie in der Finanz- und Wirtschaftskrise ergab sich beim Ausbruch der Covid-19-Pandemie 2020 erneut ein Rückgang der maßgeblichen Messgrößen für die Versichertenlöhne. Wegen der unverändert geltenden Schutzklausel folgte darauf 2021 statt einer entsprechenden Rentensenkung eine „Nullrunde“. Ohne Anwendung des Ausgleichsfaktors würde sich das Rentenniveau infolgedessen nicht nur vorübergehend, sondern anhaltend erhöhen – ausgerechnet in einer Phase stark steigender Anspannung der Rentenfinanzen aufgrund der demografischen Alterung, die mittlerweile unmittelbar bevorsteht. Die geplante Wiedereinsetzung des Ausgleichsfaktors, rechtzeitig zur Rentenanpassung 2022, sorgt somit dafür, dass der in den einschlägigen Standardregeln verankerte Status quo wiederhergestellt und der weitere Verlauf des Netto-Rentenniveaus nicht dauerhaft zugunsten der Rentner:innen bzw. zulasten der Aktiven verzerrt wird. Wegen statistischer Korrekturen ergibt sich 2022 trotzdem eine ordentliche Rentenerhöhung. Auch das Mindestniveau von 48 % würde bis 2025 aller Voraussicht nach selbst ohne Haltelinie gewahrt. Insgesamt ergibt sich eine kleine, im Rahmen des geltenden Rechts bestens vertretbare Korrektur.

Spannend ist, wie es nach 2025 weitergehen soll. Die im Koalitionsvertrag gleichfalls enthaltene Absage an längerfristig orientierte Reformen – mit Auswirkungen auf Rentenniveau und/oder Regelaltersgrenze – kann in dieser Hinsicht kaum das letzte Wort sein. Sie weckt jedoch Erwartungen, die in wenigen Jahren enttäuscht werden dürften. Auch der geplante Einstieg in eine Teilkapitaldeckung des gesetzlichen Rentensystems – zunächst mit Steuermitteln von 10 Mrd. Euro, die derzeit die Rentenausgaben für rund zehn Tage decken würden – wirft vor allem Fragen auf, etwa nach der geplanten Entwicklung solcher Reserven, nach ihrer Finanzierung aus Steuern oder Beiträgen sowie nach ihrer Umrechnung in individuelle Rentenansprüche. Die Wiedereinsetzung des Nachholfaktors ist eilig, die Entwicklung eines neuen, längerfristig orientierten Reformkonzepts für die Alterssicherung hat etwas mehr Zeit – aber besser nicht bis 2025. Bis dahin schreitet die demografische Alterung spürbar fort.

Martin Werding
Ruhr-Universität Bochum

© Der/die Autor:in 2022. Open Access: Dieser Artikel wird unter der Creative Commons Namensnennung 4.0 International Lizenz veröffentlicht (creativecommons.org/licenses/by/4.0/deed.de).

Open Access wird durch die ZBW – Leibniz-Informationszentrum Wirtschaft gefördert.
